# Coronary Flow Rate Adds Predictive Capability for FFR Assessment

**DOI:** 10.21203/rs.3.rs-2394292/v1

**Published:** 2023-01-23

**Authors:** Jacob Miller, John White, Javad Hashemi, Shahab Ghafghazi, R. Eric Berson

**Affiliations:** University of Louisville; University of Louisville; University of Louisville; University of Louisville Hospital; University of Louisville

**Keywords:** FFR, clinical prediction models, risk stratification, hemodynamics

## Abstract

A non-invasive risk assessment tool capable of stratifying coronary artery stenosis into high and low risk would reduce the number of patients who undergo invasive FFR, the current gold standard procedure for assessing coronary artery disease. Current statistic-based models that predict if FFR is above or below the threshold for physiological significance rely completely on anatomical parameters, such as percent diameter stenosis (%DS), resulting in models not accurate enough for clinical application. The inclusion of coronary artery flow rate (CFR) was added to an anatomical-only logistic regression model to quantify added predictive value. Initial hypothesis testing on a cohort of 96 coronary artery segments with some degree of stenosis found higher mean CFR in a group with low FFR < 0.8 (μ = 2.37 ml/s) compared to a group with high FFR > 0.8 (μ = 1.85 ml/s) (p-value = 0.046). Logistic regression modeling using both %DS and CFR (AUC = 0.78) outperformed logistic regression models using either only %DS (AUC = 0.71) or only CFR (AUC = 0.62). Including physiological parameters in addition to anatomical parameters are necessary to improve statistical based models for assessing high or low FFR.

## Introduction

Fractional flow reserve (FFR) is hailed as the gold standard for assessing the functional severity of a coronary artery stenosis [1]. Despite this, FFR is rarely used in clinical practices. An electronic survey determined that only 57% of cardiologists use FFR in less than one-third of cases, and that 15% of cardiologists never use FFR [2]. The low adoption rate is often accredited to associated costs, time, practicality, and potential patient complications. in response, researchers have investigated less invasive methodologies for assessing the functional severity of a coronary stenosis.

initially, Morris et al created fully transient computational fluid dynamics (CFD) models to estimate invasive FFR with a virtual counterpart (vFFR) using only angiographic images [3]. Although the model by Morris et al had excellent agreement, 96% diagnostic accuracy, it required offline computation greater than 24 hours. Papafaklis et al. developed the first protocol that required a reasonable amount of computational time. The protocol used two steady state calculations with given steady outflow boundary conditions of 1 and 3 ml/s and a constant 100 mmHg aortic pressure inlet boundary condition to produce a pressure drop versus flow rate curve. The pressure gradients from the two steady state simulations were used to calculate the Pouiselle and turbulent coefficients in the quadratic stenotic pressure drop equation. The area under the pressure gradient versus flow rate curve for a specific patient was then divided by a similar curve for an ideal artery to yield an assessment metric that was coined virtual functional assessment index (vFAi). vFAi showed excellent agreement with invasive FFR and resulted in a diagnostic accuracy, sensitivity, specificity, and AUC of 87.8%, 90.4%, 86.2%, and 0.919, respectively [4]. Morris et al. returned to their previous work to produce a less computationally expensive CFD model based on a pseudo-transient protocol that provided virtual FFR assessment in less than 4 minutes. The new pseudo-transient method produced errors less than 1% of the fully transient method that achieved 96% diagnostic accuracy [6]. Tu et al. initially created a CFD protocol for assessing FFR from 3D models generated from QCA. They calculated a volumetric flow rate using TIMI frame count which was used as an inlet boundary condition. The noninvasive assessment was eventually coined as quantitative flow ratio (QFR). The initial iteration of QFR showed good agreement with invasive FFR showing an accuracy, sensitivity, specificity, and AUC of 88%, 78%, 93%, and 0.93, respectively [5]. Some CFD models have been replaced by mathematical models which were also based on fluid dynamic principles but could be computed instantaneously. For example, QFR has been replaced with a mathematical formulation that has built upon the initial CFD protocol [7, 9–10]. There are currently three angiography-based software packages, QFR, FFR_angio_, and CAAS-vFFR, that are cleared by the Food and Drug Administration and commercially available. Of the three packages, QFR has been rigorously validated against invasive FFR with more studies underway [7–11].

Approaches which are not based on fluid dynamics have also been investigated. These methods recognize the functional-visual mismatch between FFR and coronary angiography and the factors causing the mismatch [12]. These methods, clinical prediction models, then attempt to use patient characteristics, lesion morphology, and myocardium-at-risk factors to create a scoring tool capable of stratifying the severity of an intermediate lesion. Some of these models, such as the ASLA score created by Ko et al., have used coronary computed-tomography angiography (CCTA) instead of invasive coronary angiography (ICA) with notable performance (AUC = 0.82)[13, 14]. However, most models have relied on ICA. Natsumeda et al. formulated an ICA based model which consisted of 5 independent significant predictors of FFR ≤ 0.8 which were combined to create a scoring system, STABLE, which had an AUC score of 0.76 [15]. Wong et al created the DILEMMA scoring system which used lesion length, minimum lumen diameter (MLD), and BARI MJI which is a measure of myocardium subtended by the coronary lesion [16]. The DILEMMA scoring system had an AUC of between 0.82 and 0.88 in the derivation and validation cohorts, respectively. The performance of the DILEMMA score was validated by an outside study performed by Michail et al[17].

Clinical prediction models, although not yet typically as accurate as their CFD or mathematical model counterparts, have the benefit of being simple tools that, if fully developed, can be easily adopted by clinicians and may reduce need for pressure wires [18]. Coronary flow rate (CFR) parameters have been generally excluded from these clinical prediction models despite the cornerstone publications of Gould establishing that the functional severity of an intermediate stenosis is highly dependent on the flow rate through the artery [19]. Flow disturbance after the stenosis, which is related to pressure drop and other non-pressure based metrics for severity, is also known to be a function of flow rate [20]. Therefore, it is not known how including CFR affects the performance of a clinical prediction model.

The purpose here was to determine if including CFR added predictive value to clinical prediction models, particularly compared to percent diameter stenosis (%DS), the most used parameter for percutaneous coronary intervention (PCI) decision. This was met through three specific aims. First, determine if there is a significant statistical difference in CFR between low (FFR ≤ 0.8) and high (FFR > 0.8) groups. Second, determine if including CFR in a predictive model improves accuracy, sensitivity, and specificity relative to a model only containing anatomical features such as %DS. Third, provide a causal explanation for the relationship between CFR and FFR by visualizing differences in hemodynamic flow features between low and high patients using CFD.

## Methods

### Patient Population

One hundred arteries from ninety patients who underwent invasive coronary angiography and FFR measurements in two affiliated hospitals were considered for this study. The inclusion criteria for this study were all patients with stenoses in major epicardial arteries such as RCA, OM, LCX, or LAD. The exclusion criteria were ostial lesions in the left main artery or RCA, bifurcations, and lesions distal to bypass grafts. Following the application of the exclusion criteria, ninety-six patients were suitable for retrospective investigation. The Institutional Review Board at the University of Louisville approved the study protocol used for patient cases and all research was performed in accordance with relevant regulations. As the study was retrospective, and non-interventional in nature with minimal risk to subjects, the IRB granted waiver of informed consent.

### 3D Rendering and Angiographic Feature Extraction

The CAAS 7.5 QCA-3D workstation (Pie Medical Imaging, Maastricht, The Netherlands) was used to generate 3D renderings given two angiographic images taken 30° apart [4]. Output from the workstation was used to catalog or calculate mean lumen area, minimum lumen area, minimum lumen diameter, and length of arterial segment. Minimum lumen diameter and mean lumen diameter were calculated from minimum lumen area and mean lumen area by assuming a perfect circle equal to the respective areas. Percent diameter stenosis was defined as the difference between unity and the minimum lumen diameter divided by the maximum lumen diameter of a given arterial segment.

### Meshing, CFD, and Coronary Flow Rate Determination

Computational fluid dynamics modeling of coronary blood flow was performed with ANSYS Fluent 17.0. The viscous model was set to laminar, and density was set as a constant value of 1045 kg/m^∧^3. A 100% hematocrit value was assumed to have a viscosity equal to 0.008 kg m-1 s-1, and a 0% hematocrit was assumed to have a viscosity equal to 0.001 kg m-1 s-1. Linear interpolation between these two extrema were used to find the patient viscosity values. Patient viscosities ranged from 0.0035 to 0.0045 kg m-1s-1, and it should be noted that small changes in viscosity did not appreciably affect the calculations. The 3D renderings provided by the CAAS 7.5 QCA-3D workstation were used to create unstructured computational meshes. The unstructured computational meshes consisted of tetrahedral cells with 10 inflation layers. A mesh sensitivity analysis was performed which determined the optimum cell count of 542,000 for an arterial segment with a total lumen volume equal to 4.04 × 10^∧^−8 m^∧^3. The node count was scaled accordingly for arterial segments of different total lumen volumes. CFR through the arterial segment was determined by iteratively varying a volumetric flow rate boundary condition until the virtual FFR value was within 1% of the invasive FFR value.

### Statistical Analysis

Continuous variables are reported as means ± one standard deviation (SD) or median (interquartile range, 25th-75th percentile) for abnormal distributions. Categorical variables are represented as percentages. Variables were investigated visually using histograms and quantile-quantile (qq) plots. All parameters were tested for normality using the Shapiro-Wilk test. Scatter plots were created to visualize the relationship between variables of interest and FFR. The strength of the linear relationship was quantified using Pearson correlation coefficients. Two sample t-tests and Mann-Whitney U-tests were used to test mean differences between FFR < 0.8 and FFR > 0.8 groups after testing for normality. Logistic regression models from the python package, StatsModels, were used to map input features from ICA and CFD to the binary outcome of FFR ≤ 0.8 or FFR > 0.8. The difference in goodness-of-fit between nested logistic regression models was quantified using the log-likelihood test for significance. The predictive capability of a model was compared to that of others by various metrics including accuracy, sensitivity, specificity, positive predictive value, negative predictive value and area-under-the-curve (AUC) values. Model performance was compared using receiver operator characteristic (ROC) curves. Odds ratios of the model coefficients were used as measures of effect size for the variables of interest.

## Results And Discussion

Pressure drop through a constriction is expressed as the product of flow rate through the geometry, Q, and the total fluidic resistance, R, in the hydraulic resistance equation (Equation 1). Therefore, a stenotic risk model which includes the flow rate and fluidic resistance should better predict the pressure drop across the vessel when compared to single parameter models.


(1) ΔP=QR

To investigate if coronary flow rate, CFR, would improve a risk scoring tool, we began by performing hypothesis testing to determine if there was a significant difference in mean CFR between the FFR < 0.8 and FFR > 0.8 groups. A Mann-Whitney U-Test determined that there was a significant difference in CFR between FFR ≤ 0.8 (μ=2.37 ml/s) and FFR > 0.8 (μ =1.85 ml/s) groups (p-value=0.046). The statistically significant difference in mean coronary artery flow between the two groups indicates that CFR may be an important predictor in coronary artery disease. To highlight the already known importance of %DS, a two-sample t-test showed there was a significant difference in %DS among FFR ≤ 0.8 (μ =54%) and FFR > 0.8 (μ =46%) groups (p-value= 0.0001).

**Figure 1:**
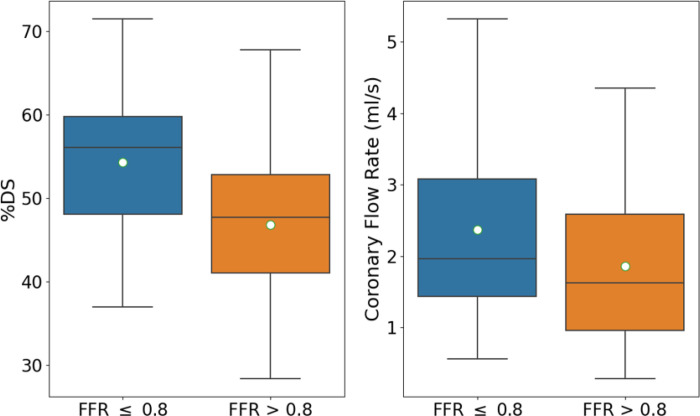
Box plot of %DS and coronary flow rate for FFR ≤ 0.8 and FFR > 0.8 patient groups. Mean CFR for FFR ≤ 0.8 and FFR > 0.8 is 2.37 ml/s and 1.85 ml/s, respectively. Median CFR for for FFR ≤0.8 and FFR > 0.8 is 1.96 ml/s and1.63 ml/s, respectively. Mean %DS for FFR ≤0.8 and FFR > 0.8 is 54% and 46%, respectively. Median CFR for for FFR ≤0.8 and FFR > 0.8 56% and 48%, respectively.

Conclusions from hypothesis testing is further supported by a box plot which shows the means (denoted by the white dots), medians, and spread of the data (Figure 1). As expected, the FFR ≤ 0.8 group had a greater mean (54% vs 46%) and median (56% vs 48%) %DS than the FFR > 0.8 group. The novel finding is the difference in CFR between FFR ≤ 0.8 and FFR > 0.8 groups, specifically that the FFR ≤ 0.8 group had a higher mean (2.37 ml/s vs 1.85 ml/s) and median (1.96 ml/s vs 1.63 ml/s) CFR than the FFR > 0.8 group. To give a specific example, two patients whose %DS differed by less than 1% differed in FFR by 0.33. The high FFR patient had a CFR half that of the low FFR patient. Increased CFR through a constriction increases distal Pouiselle frictional losses and turbulent dissipation, increasing the pressure loss, and leading to a lower FFR. While CFR is ordinarily reduced through a stenosed segment because of a severe blockage, low FFR patients may already have had relatively high CFR prior to the formation of the blockage. The correlation between high CFR and low FFR suggests the possibility that already high CFR is causal to the formation of more severe stenoses.

To further investigate the effect of CFR on FFR, logistic regression models were created to study binary classification ability. Table 1 summarizes the performance metrics of three different logistic regression models: %DS only model, CFR only model, and a parent model containing both %DS and coronary flow rate as input features. Accuracy, sensitivity, and specificity were 56%, 45%, and 67% in the CFR only model. Although there is a significant difference in mean CFR between FFR ≤ 0.8 and FFR > 0.8 groups, CFR by itself is about the same as random chance at predicting functionally significant stenoses. %DS by itself was only marginally better at predicting functionally significant coronary lesions with an accuracy, sensitivity, and specificity of 62%, 62%, and 63%.

To determine if including CFR as an input feature into the %DS model results in a significant improvement in predictive capability over the individual models, a log-likelihood test was performed [16]. The log-likelihood test resulted in a p-value of 0.005 which verifies a statistically significant difference in predictive capability between the %DS model only and the combined parent model.

Table 2 summarizes the significance of each input feature within the parent model. The odds ratio (OR) of the %DS shows that there is a 13% increase in the odds of having a functionally significant stenosis for every 1% increase in %DS. The odds ratio of CFR shows that there is 103% increase in odds of having a functionally significant stenosis for every 1 ml/s increase in CFR. The large increase in odds is likely due to the magnitude of the unit increase in coronary flow rate relative to the typical coronary flow rate. The range of coronary flow rates included in this study ranged from less than 1 ml/s to 5 ml/s. Therefore, a 0.25 ml/s increase in even the largest flow rate at a given %DS would amount to an increase of 25.75% in coronary flow rate. Regardless, investigation of the parent model by inspecting odds ratios of the input features adds further evidence that flow rate and %DS are both important for predicting functionally severe lesions.

**Figure 2:**
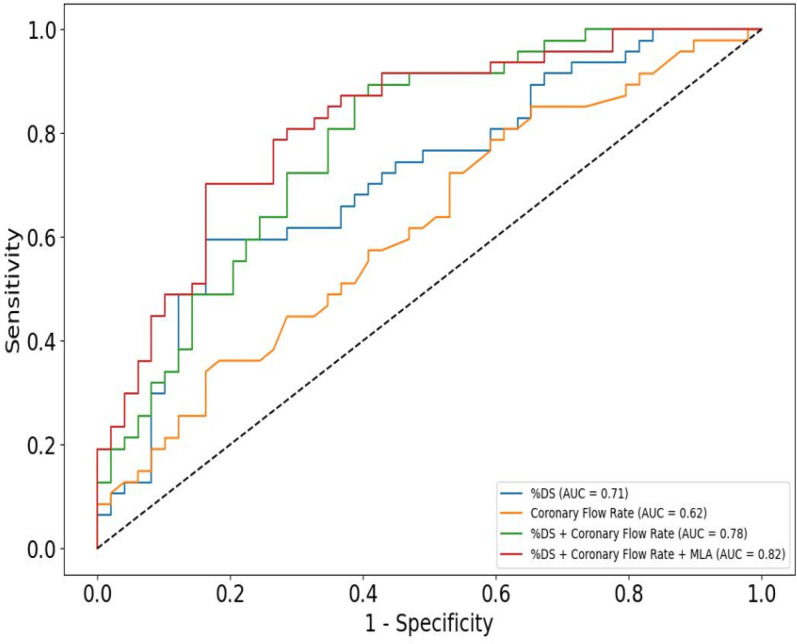
ROC curves of various predictive models using coronary flow rate and angiographic parameters.

Figure 2 shows ROC curves and AUC values for the %DS model, the CFR model, and the combined %DS- CFR parent model. The single parameter CFR model performed the worst with an AUC of 0.62. The single parameter %DS model performed better with an AUC value of 0.71. The largest increase in model performance came from including both CFR and %DS in a single model which increased AUC to 0.78, supporting the claim that CFR incrementally improves FFR predictive capability over %DS alone. MLA was included as an angiographic feature that differentiates between cases with similar %DS. A given CFR through a larger MLA but same %DS will not create the same recirculation when exiting the stenosis. Adding minimum lumen area (MLA) to the parent model increased model performance slightly to an AUC value of 0.82.

**Figure 3:**
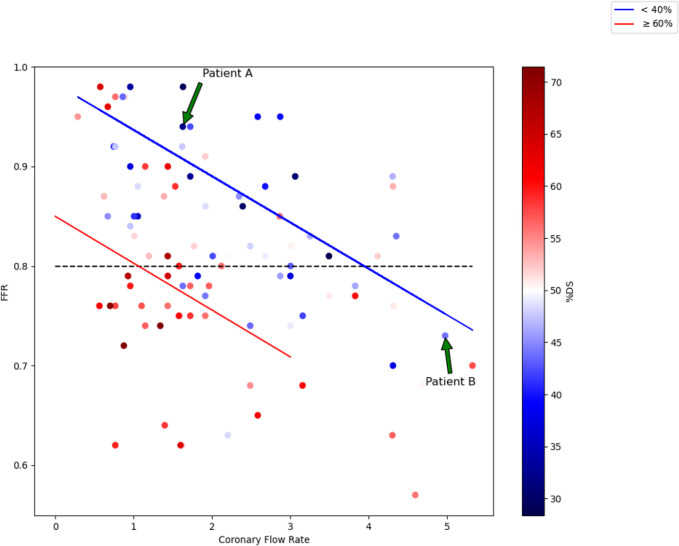
Scatter plot of FFR versus CFR and colored by %DS. The blue line represents the line-of-best-fit for patients with a %DS less than 40%. The red line represents the line-of-best-fit for patients with a %DS greater than or equal to 60%. The dashed black line indicates the clinical threshold for significance.

A scatter plot of FFR versus CFR colored by %DS (Figure 3) quantifies the effect of %DS and CFR on FFR. The black dashed line represents the clinically accepted threshold for stenosis severity (FFR < 0.8). The red line is a line of best fit for patients which had an %DS ≥ 60%, and the blue line is a line of best fit for patients which had an %DS less than 40%. There is a higher density of less severe stenoses (dark blue dots) above the clinical threshold, and a higher density of high grade stenoses (dark red dots) below the clinical threshold. The negative slopes of the trend lines indicate that increases in CFR will lower the FFR for both low and high %DS. The relative positions and the y-intercepts of the lines indicate FFR is generally higher for lower %DS at a given flow rate. More interestingly, Figure 3 provides explanations for cases which are seemingly unexpected, such as those that have a low %DS and low FFR. These cases generally have a higher CFR. There are also examples of very high %DS patients that have some of the highest FFR values in the entire cohort. The pressure losses through these stenoses are small due to the low hyperemic flow rate, which limits the Pouiselle and turbulent losses. There are a few cases that have FFR ≤ 0.8 values at both mid-to-low %DS and mid-to-low CFR. These patients may have other anatomical features which would lead to lower FFR, such as severely asymmetric stenoses which would increase turbulent losses, diffuse lesions which would increase Pouiselle losses, or microvascular disease

The data points with high FFR and high %DS or vice versa seen in Figure 3 support the well-established result that anatomical assessment alone is not sufficient to correctly diagnose the clinical significance of a stenosis. The importance of CFR in explaining FFR is highlighted when comparing two patients with similarly low %DS, but one with low CFR and one with high CFR (labeled Patient A and Patient B in Figure 3). Patients A and B both have low %DS (44% and 32%), but Patient A has a much lower CFR (1.63 mL/sec) compared to Patient B (4.97 mL/sec). Patient A (low %DS / low CFR) has an FFR = 0.94 (well above the threshold) while Patient B (low %DS / high CFR) has an FFR = 0.73 (well below the threshold). In terms of Equation 1, the resistance term, R, is similar for both while the flow rate term, Q, for Patient B is 3 times that of Patient A, creating a larger pressure drop and, hence, lower FFR.

**Figure 4:**
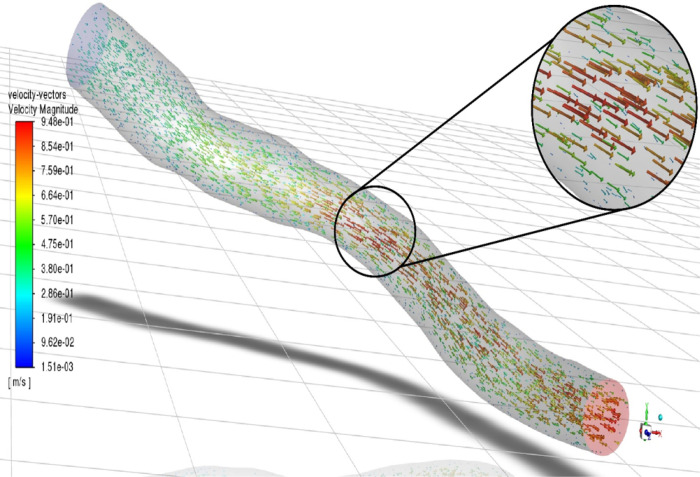
Velocity vector colored by velocity magnitude for Patient A (Low %DS and Low FR). Flow remains well ordered distal to the stenosis.

**Figure 5:**
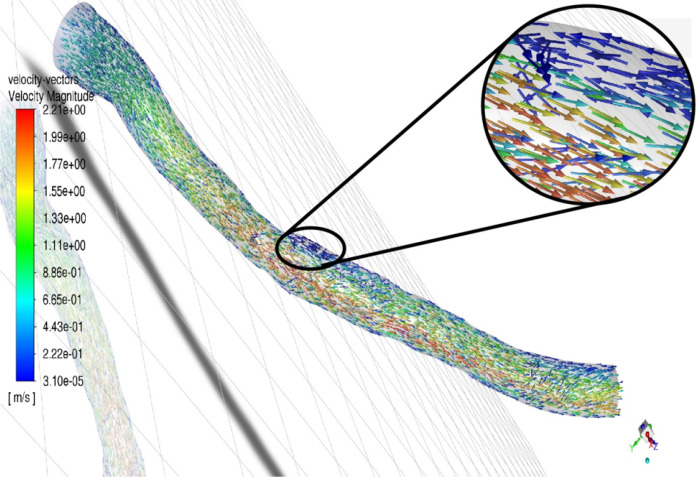
Velocity vector colored by velocity magnitude for Patient B (Low %DS and High FR). A recirculation region forms distal to the stenosis.

The flow features of Patients A and B are visualized in Figure 4 (Patient A) and Figure 5 (Patient B) as vectors of velocity colored by velocity magnitude. Flow distal to stenosis remains well ordered for Patient A. However, the higher flow rate for Patient B creates a region of flow reversal due to the adverse pressure gradient formed by the expansion and acceleration of fluid exiting the stenosis (zoomed region of Figure 4). This flow reversal causes the frictional and turbulent energy losses responsible for the larger pressure drop. The flow reversal and recirculation has been quantified in terms of higher residence times, which correlate to lower FFR [20].

### Limitations

If the estimated CFR values used in this study differ significantly from actual CFR values, then this could affect regression coefficients which would change the odds ratios. However, the CFR used in this study produced a virtual FFR computed within 1% of invasively measured FFR for each case, providing evidence for validity of the results here. Direct measurement of coronary flow, such as by angiographic contrast [22], is feasible, but these techniques are invasive and time consuming. Measuring coronary flow rates in vivo would provide experimental validation of the correlation between FFR and coronary flow rates. Regression and machine learning type methods, when fully developed, could be used to estimate volumetric flow rate from anatomical imaging and other readily available patient data. Future work will address the best strategy to determine inlet volume flow rate.

## Conclusion

This study found that including CFR in stenotic risk models results in greater predictive performance than models which only include angiographic parameters. We have shown the theoretical significance of CFR through Equation 1, the clinical significance of CFR through statistical analysis, and the underlying causal mechanisms for the effect of CFR on FFR through CFD. The greater predictive capability of the %DS-CFR logistic regression model over either the %DS-only or CFR-only model supports the relationship in Equation 1 between stenotic resistance and stenotic flow rate to accurately describe pressure losses. The large odds ratio of CFR highlights the importance of CFR in predicting FFR. Lastly, the flow disturbance distal to stenoses, which was visualized by fluid modeling in Figure 5, provides a causal explanation for the significance of CFR in predicting FFR that was first alluded to by Equation 1 and then validated statistically using logistic regression. We recommend that existing and new clinical prediction models for coronary artery stenosis severity incorporate physiological flow rate that could be obtained from established measurement techniques. Improved predictive capability may result in improved risk stratification tools for coronary artery disease thereby saving time, cost, and unnecessary intervention.

## Figures and Tables

**Table 1.** Performance metrics of logistic regression models for %DS, coronary flow rate, and a parent model containing both.

	%DS Only	Flow Rate Only	Parent Model
Accuracy	62%	56%	71%
Sensitivity	62%	45%	70%
Specificity	63%	67%	71%
Positive Predictive Value	62%	57%	70%
Negative Predictive Value	63%	56%	71%

**Table 2:** Summary of the odds ratios, confidence intervals and p-values of the parent model.

Input Feature	OR	Lower 95% CI	Upper 95% CI	P-values
constant	4.480E-4	1.16E-5	0.0165	0.000
%DS	1.13	1.06	1.20	0.000
Coronary Flow Rate	2.03	1.29	3.22	0.002

## Data Availability

The datasets used and/or analyzed during the current study available from the corresponding author on reasonable request.
